# The mystery behind dark-orange tomatoes: How can an exotic cleavage dioxygenase alter carotenoid flux?

**DOI:** 10.1093/plphys/kiad164

**Published:** 2023-03-21

**Authors:** Lara Pereira

**Affiliations:** Plant Physiology, American Society of Plant Biologists, USA; Ecology and Evolutionary Biology, School of Biosciences, University of Sheffield, Sheffield S10 2TN, UK

Carotenoids are members of a large and highly diverse family of natural products, called isoprenoids, that are synthesized primarily in plants. The oxidative cleavage of carotenoids results in the formation of another important class of molecules, the apocarotenoids. Together, carotenoids and apocarotenoids are essential for photosynthesis and photoprotection and as hormone precursors, and they play myriad roles in plant development and signaling (Hou et al. 2016). Carotenoids are also vital nutrients for humans, acting as vitamin precursors, antioxidants, and antitumoral compounds, and they contribute to the pleasant aromas and flavors associated with many ripe fruits.

**Figure 1.**
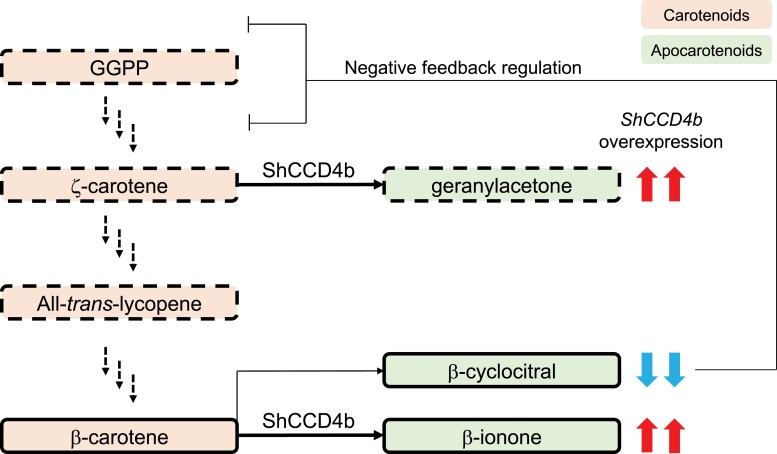
Enzymatic activity of ShCCD4b in tomato. Simplified representation of the carotenoid and apocarotenoid pathways (modified from Yoo et al., 2023). Only relevant metabolites are displayed, and intermediate metabolites and enzymatic steps have been omitted for clarity. Dashed borders are used for acyclic metabolites, and solid borders are for cyclic metabolites. In *ShCCD4b*-overexpressing lines, apocarotenoids are either more abundant (red arrows) or less abundant (blue arrows).

In tomato (*Solanum lycopersicum*), crop improvement has severely deteriorated fruit flavor and consumer satisfaction (Tieman et al. 2017). Very low levels of apocarotenoids confer a sweet and floral odor that improves the overall liking of tomato fruits by consumers (Vogel et al. 2010). Therefore, during the last few decades, there has been increased interest in understanding the genetic basis of the metabolic profiles of tomato fruits with the goal of applying this knowledge toward breeding programs aimed at restoring lost flavor to this important vegetable crop.

Apocarotenoid biosynthesis depends on the activity of carotenoid cleavage dioxygenases (CCDs) (Hou et al. 2016). Nine families of CCDs have been described in Arabidopsis (*Arabidopsis thaliana*), and they present variable substrate specificity and cleavage sites (Moreno et al. 2021). Some of these enzymes have been characterized in several plant species, showing a degree of variation in the resulting apocarotenoids.

In this issue of *Plant Physiology*, Yoo et al. (2023) characterized an exotic *Solanum habrochaites* allele of *CCD4b* (*ShCCD4b*) using a multidisciplinary approach that included classical genetics, biotechnology, and metabolomics. They cloned and validated the responsible gene, characterized its enzymatic activity, and proposed a hypothesis about the feedback regulation of carotenoid flux in tomato.

Initially, the authors identified the responsible genetic factor for dark-orange fruit in an introgression line by positional cloning, reaching a narrow candidate region that contained 2 *CCD* genes, *CCD4a* and *CCD4b*. Only *CCD4b* was differentially expressed in ripe fruits, and its expression in dark-orange fruits from the introgression line was ∼8,000-fold higher than in cultivated tomatoes.

The dark-orange phenotype, caused by reduced content of *trans*-lycopene, β-carotene, and lutein, was recreated in *ShCCD4b*-overexpressing lines. The metabolic profiles of ripe fruits from these transgenic lines were altered, and the relative levels compared to cultivated tomato, together with enzymatic essays in *Escherichia coli*, allowed the authors to elucidate the enzymatic activity of CCD4b. CCD4b cleaved β-carotene and ζ-carotene at position C9-C10 (C9′-C10′), and the products of this reaction, β-ionone and geranylacetone, were detected at higher levels in *ShCCD4b*-overexpressing lines (Figure 1). In contrast, in other species, CCD4 is unable to cleave acyclic *cis*-carotenoids such as ζ-carotene (Rubio et al. 2008; Bruno et al. 2016).

Carotenoid flux is thought to occur under a feedback regulation controlled by apocarotenoids derived from either acyclic *cis*-carotenes (Moreno et al. 2021) and/or β-carotene (Hou et al. 2016). In this study, carotenoid profiles in the fruits were not associated with the enzymatic activity, indicating that the carotenoid flux was altered in *ShCCD4b*-overexpressing lines. Surprisingly, Yoo et al. (2023) proposed β-cyclocitral as the main negative feedback regulator. Classically, the apocarotenoid signal regulating carotenoid flux was thought to be an acyclic apocarotenoid (Kachanovsky et al. 2012; Hou et al. 2016), but Yoo et al. (2023) showed that, even though acyclic carotenoids do act as CCD4b substrate, the signaling molecule is not the linear geranylacetone but the cyclic carotenoid derived from β-carotene.

*ShCCD4b*-overexpressing lines are deficient in β-cyclocitral, presumably because available β-carotene is preferentially transformed into β-ionone by CCD4b (Figure). The resulting reduction in β-cyclocitral promotes carotenoid biosynthesis in tomato, increasing the amount of carotenoids and apocarotenoids, as already shown in Arabidopsis (Mitra et al. 2021). To further validate the hypothesis that postulates β-cyclocitral as the apocarotenoid signal regulating the negative feedback loop, several apocarotenoids were exogenously applied to tomato and bell pepper (*Capsicum annuum*) fruits. Only β-cyclocitral inhibited carotenoid synthesis, while β-ionone and geranylacetone did not affect the carotenoid flux. In fact, in *ShCCD4*-overexpressing lines, ζ-carotene levels were elevated despite being a substrate of CCD4b, which suggests that the increment in the carotenoid flux is higher than the turnover rate of geranylacetone from ζ-carotene.

Phytoene synthase 1, the first, rate-limiting enzymatic step of the carotenoid pathway, has been proposed to be the target of both transcriptional and posttranscriptional regulation by apocarotenoids (Moreno et al. 2021). In Yoo et al. (2023), expression levels of all biosynthetic genes in the pathway were quantified, but no clear association between gene expression and the amount of β-cyclocitral was found in the *ShCCD4b*-overexpressing lines nor in the fruits treated with exogenous β-cyclocitral. Therefore, the step of the pathway at which feedback regulation by β-cyclocitral is executed remains to be elucidated.

To conclude, the exotic allele *ShCCD4b* can easily be integrated into the breeding pool by using molecular markers, consequently increasing carotenoid and apocarotenoid amounts. Since apocarotenoids have a huge positive impact in flavor perception without affecting yield, this strategy offers an optimal solution to restore tasty tomatoes while keeping both consumers and the industry satisfied.
